# Automatic caries detection in bitewing radiographs—Part II: experimental comparison

**DOI:** 10.1007/s00784-024-05528-2

**Published:** 2024-02-05

**Authors:** Antonín Tichý, Lukáš Kunt, Valéria Nagyová, Jan Kybic

**Affiliations:** 1 Institute of Dental Medicine, First Faculty of Medicine of the Charles University and General University Hospital in Prague, Prague, Czech Republic; 2 Faculty of Electrical Engineering, Czech Technical University in Prague, Prague, Czech Republic

**Keywords:** Dental caries detection, Convolutional neural networks, Ground truth, Bitewing, X-ray images

## Abstract

**Objective:**

The objective of this study was to compare the detection of caries in bitewing radiographs by multiple dentists with an automatic method and to evaluate the detection performance in the absence of a reliable ground truth.

**Materials and methods:**

Four experts and three novices marked caries using bounding boxes in 100 bitewing radiographs. The same dataset was processed by an automatic object detection deep learning method. All annotators were compared in terms of the number of errors and intersection over union (IoU) using pairwise comparisons, with respect to the consensus standard, and with respect to the annotator of the training dataset of the automatic method.

**Results:**

The number of lesions marked by experts in 100 images varied between 241 and 425. Pairwise comparisons showed that the automatic method outperformed all dentists except the original annotator in the mean number of errors, while being among the best in terms of IoU. With respect to a consensus standard, the performance of the automatic method was best in terms of the number of errors and slightly below average in terms of IoU. Compared with the original annotator, the automatic method had the highest IoU and only one expert made fewer errors.

**Conclusions:**

The automatic method consistently outperformed novices and performed as well as highly experienced dentists.

**Clinical significance:**

The consensus in caries detection between experts is low. An automatic method based on deep learning can improve both the accuracy and repeatability of caries detection, providing a useful second opinion even for very experienced dentists.

## Introduction

With more than 3.5 billion people affected, dental caries is the most prevalent disease [1, 2]. While preventive measures are considered as the primary way to decrease the dental care expenses, early caries detection is also important, as it may avoid the need of costly restorative treatment [3]. However, the widely used visual inspection or visual-tactile examination may be insufficient to detect incipient caries [4, 5]. In particular, this applies to the proximal surfaces of posterior teeth, for which radiographs are frequently taken [6].

According to a systematic review and meta-analysis by Schwendicke et al. [7], radiographic caries detection is highly accurate for cavitated lesions and dentin caries. However, lower sensitivity was found for initial lesions, and it was suggested that other complementary methods, such as laser fluorescence, transillumination, or electric conductivity measurement [8], are used in a population with high caries risk and prevalence. The meta-analysis also reported a high variability in accuracy and low-inter observer agreement [7, 9]. The underlying factors of the variability were classified as clinical (e.g., lesion depth, dentition, surface location) and methodological (e.g., clinical vs. in vitro settings, reference standard, the number and experience of examiners) [7]. Some in vitro studies reported high inter- and intra-observer agreement [10, 11]. However, the in vitro assessment is considerably different from clinical in vivo studies. As a result, in vitro studies might overestimate sensitivity and underestimate specificity. They were also reported to be more susceptible to small-study effects or publication bias [7].

### Deep learning

It has been suggested that deep learning could assist in overcoming some of the mentioned issues. Convolutional neural networks (CNNs) have been used in various medical applications, including dental caries detection. In many tasks, e.g., classification, detection, or segmentation, the performance of CNNs is comparable or even superior to experts [9, 12]. For caries detection, image datasets are annotated by experts and the labeled data are then used for the training of CNNs which learn to recognize specific features of caries. Provided that the dataset has a sufficient quality and size, CNNs are able to predict caries in unknown images with a high accuracy [9, 12].

The annotation requires a high level of expertise and is very time-consuming. Furthermore, the ground truth should preferably be based on the opinion of multiple experts, as the reference set by a single expert may be biased [9]. On the other hand, if the dataset is annotated by multiple experts, the inter-expert variability may lead to incongruous annotations. This problem may be mitigated by using majority voting, but in the absence of a solid reference, visual evaluation of the radiographs should not be regarded as fully conclusive.

The reference standard, also called the “gold” standard, may be destructive (histologic, microradiographic or operative assessment) or non-destructive (visual-tactile assessment) [7]. Given the high number of images required for machine learning, destructive methods are not applicable. Therefore, three of the previous studies [13–15] verified the existence of caries clinically but that may have even been counterproductive, given the low sensitivity of proximal caries detection in posterior teeth [5]. The uncertainty led some researchers to use a 5-point scale: 1, caries definitely present; 2, caries probably present; 3, uncertain-unable to tell; 4, caries probably not present; and 5, caries definitely not present [10, 11, 16].

### Experimental evaluation

The first objective of this work was to compare the performance of a deep learning-based automatic caries detection method presented in a companion “Part I” paper [17] to 8 human annotators, ranging from novices to experts, and including the original annotator who created the training dataset for the automatic method. The second objective was to address the unavailability of the “gold” standard for reference. Multiple ways of evaluating the performance were used, including pairwise comparisons and creation of a consensus standard. The methods are first described in “Methods” section with most results shown in “Results” section.

## Methods

The best performing method from “Part I” [17] was used. It is an ensemble of 4 different types of object detection CNNs: RetinaNet-SwinT, Faster R-CNN-ResNet50, YOLOv5-M and RetinaNet-R101. The automatic method, denoted *M*, was trained on a dataset $D_0$ with 3989 anonymized bitewing images [17]. The carious lesions were annotated by tight fitting bounding boxes by an expert $E_0$ with 5 years of experience (A.T.) The Computer Vision Annotation Tool (CVAT)^1^ was used for annotations.

**Fig. 1:**
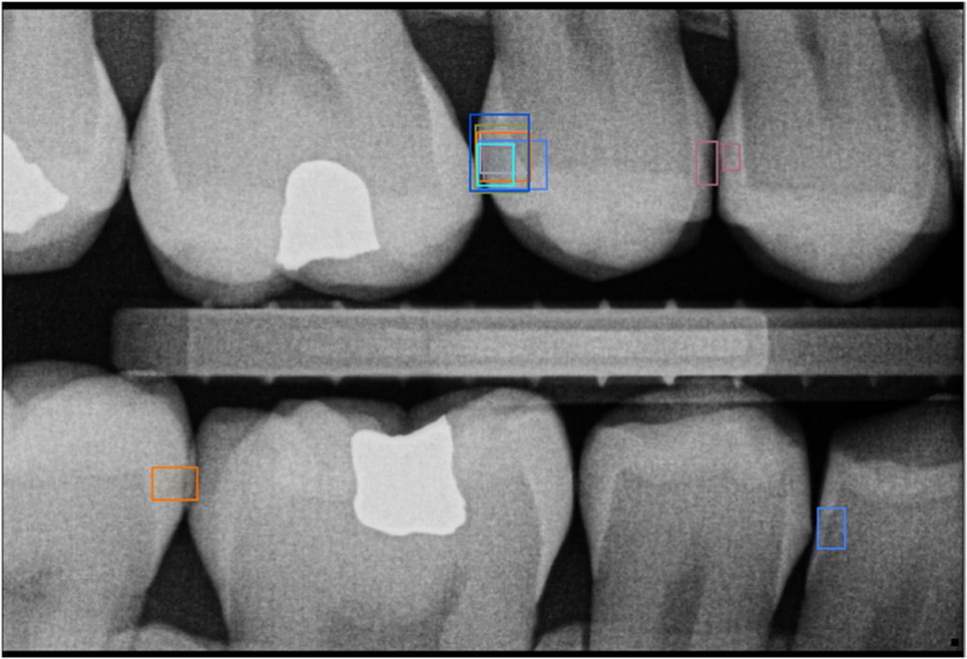
Sample image from $D_1$ with the annotations of the 8 human annotators. Each color corresponds to one annotator

For testing, *dataset* $D_1$ containing 100 images was created [18] with no overlap between $D_0$ and $D_1$. As in $D_0$ [17], the radiographs in $D_1$ were acquired using four different intraoral X-ray units, three of which used direct radiography and one employed indirect radiography. Sensor physical dimensions ranged from $31\times 41$ mm to $27\times 54\,\text {mm}$. To simplify processing, all images were rescaled to $896\times 1024$ pixels, with the wide-sensor images padded with black horizontal margins to preserve the aspect ratio. Bitewings with large overlaps of adjacent proximal surfaces or major artifacts were excluded from $D_1$. Bitewings in $D_1$ presented only permanent teeth, but their inclusion was not limited by the number of displayed teeth, presence or size of caries and presence of restorations.

Besides $E_0$, four dentists with more than fifteen years of experience (*experts*, denoted $E_1,\dots ,E_4$) and three dentists with less than five years of experience (*novices*, denoted $N_1,N_2,N_3$) were recruited. The dentists were given instructions on how to use CVAT and asked to annotate all carious lesions in dataset $D_1$ regardless of their size using tight fitting boxes. The annotators worked completely independently in order to avoid introducing any bias.

The group of all annotators will be denoted $\textsf {A}=\bigl \{E_0,E_1,\dots ,E_4,N_1,N_2,N_3,M\bigr \}$, including the automatic method *M*. For each image $i\in D_1$, each annotator $a \in \textsf {A}$ yielded a (possibly empty) set of detections, represented as bounding boxes $\textsf {B}_{ia}=\bigl \{b_{ia}^1,b_{ia}^2,\dots \bigr \}$. Example annotations of the same image (Fig. 1) show that there were marked differences between annotators in both the size and position of bounding boxes. This was confirmed by the annotation statistics in Table 1 — the number of annotations varied between 241 and 425, and one annotator ($E_4$) created bounding boxes twice as big as most of the others.

**Table 1:** Number of annotations, mean length of rectangle sides and their standard deviation for each annotator on dataset $D_1$

Annotator	Num	Mean	Std
*M*	256	47.96	21.95
$E_0$	269	54.76	25.13
$E_1$	425	65.37	31.92
$E_2$	264	50.91	30.34
$E_3$	294	74.03	32.77
$E_4$	241	100.11	35.98
$N_1$	384	56.53	21.92
$N_2$	366	62.93	24.13
$N_3$	342	57.88	30.29

### Pairwise comparison

The similarity of the annotations between all pairs of annotators $(a,a')\in \textsf {A}\times \textsf {A}$ was evaluated. For each image *i*, two sets of bounding boxes, $\textsf {B}_{ia}$ and $\textsf {B}_{ia'}$ were produced, which will be denoted $\textsf {B}$ and $\textsf {B}'$, respectively.

Two bounding boxes *b* and $b'$ were considered to correspond to the same lesion if the centroid of one was inside the other or vice versa

1 $$\begin{aligned} b \cong b' \iff \text {centroid}(b) \in b' \vee \text {centroid}(b') \in b \end{aligned}$$

Note that this relation is reflexive and symmetric but not transitive.

To evaluate the similarity between the two sets of annotations $\textsf {B}$ and $\textsf {B}'$, we first found a matching $\Omega \subseteq \textsf {B} \times \textsf {B}'$, such that all pairs $(b,b')\in \Omega$ matched ($b \cong b'$) and each box from $\textsf {B}$ or $\textsf {B}'$ appeared in $\Omega$ at most once. The correspondence was usually rather clear, so the following simple greedy algorithm was used:

1. Find the largest box *b* from $\textsf {B} \cup \textsf {B}'$. Without loss of generality, assume that $b\in \textsf {B}$, otherwise exchange the roles of *b* and $b'$.
2. Find a corresponding box $b' \in \textsf {B}'$ such that $b'\cong b$ (see (1)), i.e., the boxes match. If there are multiple such $b'$, choose the one that maximizes the intersection $\bigl |b \cap b'\bigr |$. If it is not unique, pick the largest $b'$.
3. If a match $b'$ was found, insert $(b,b')$ into $\Omega$ and remove *b* from $\textsf {B}$ and $b'$ from $\textsf {B}'$.
4. Repeat until $\textsf {B}$ or $\textsf {B}'$ is empty or all boxes have been considered.

The *number of errors* for the current image *i* was then the number of remaining unmatched boxes

2 $$\begin{aligned} e^i_{aa'}= e(\textsf {B},\textsf {B}')=\bigl |\textsf {B}\bigr |+\bigl |\textsf {B}'\bigr | \end{aligned}$$

Both missed lesions (false negatives) and incorrect detections (false positives) were counted as errors. The total number of errors for two annotators *a*,$a'$ was the sum over all images

3 $$\begin{aligned} e_{aa'}=\sum _{i \in D_1} e^i_{aa'} \end{aligned}$$

The number of errors is important, because it indicates the agreement of the annotators on the presence or absence of caries in a certain part of the tooth, irrespective of the pixel-precise location and size of the bounding box that differed widely among annotators. This measure was introduced to evaluate the annotation agreement by other means than the widely used *intersection over union* (IoU), which often reaches low values even when it is clear that the same lesion is annotated.

Mean IoU was subsequently calculated to evaluate the overlap of the matched bounding boxes between two annotators *a*, $a'$ over the whole dataset as a mean of all matched annotations

4 $$\begin{aligned} \text {IoU}_{aa'}= \frac{\displaystyle \sum _{i\in D_1} \sum _{(b,b')\in \Omega _i^{aa'}} \text {IoU}(b,b')}{\displaystyle \sum _{i\in D_1} \bigl | \Omega _i^{aa'} \bigr |} \end{aligned}$$

where $\Omega _i^{aa'}$ was the identified matching between annotations of *a* and $a'$ in image *i*. $\text {IoU}_{aa'}$ served to evaluate the localization accurracy, while ignoring unmatched annotations, including completely missed (false negative) or spurious (false positive) annotations. These were only reflected in the number of errors $e_{aa'}$.

### Significance of pairwise differences

For the pairwise comparison with experts $\bigl \{E_0,E_1,E_2,E_3,E_4\bigr \}$, the significance of the differences between annotators *a* and *b* in terms of the number of errors *e* was evaluated by the Wilcoxon signed-rank test applied to the sequence

5 $$\begin{aligned} \Delta e^i_{ab}=\sum _{\begin{array}{c} c\not =a\\ c\not =b \end{array}} e(\textsf {B}_{ia},\textsf {B}_{ic})-e(\textsf {B}_{ib},\textsf {B}_{ic}) \end{aligned}$$

where the sum was over the experts, $c\in \bigl \{E_0,E_1,\dots ,E_4\bigr \}$. An analogous procedure was performed for the IoU measure. It is noteworthy that the non-expert annotators including *M* were disadvantaged in these comparisons, as they were not used as a reference. For results, see “Pairwise comparison” section.

### Average number of errors and IoU

The measures $\text {IoU}_{aa'}$ and $e_{aa'}$ for a given annotator *a* were averaged over either experts ($a'\in \bigl \{E_0, E_1,E_2,E_3,E_4\bigr \}$) or over all other annotators excluding *M* to evaluate how close each annotator is to the “human average”:

6 $$\begin{aligned} \text {IoU}_a&={\text {mean}}_{a'\not =a} \text {IoU}_{aa'} \end{aligned}$$

7 $$\begin{aligned} e_a&={\text {mean}}_{a'\not =a} e_{aa'} \end{aligned}$$

Note that this definition disadvantaged *M*, which was never included in the mean.

### Comparison with a consensus standard

As an alternative to the pairwise evaluation described above, the annotations of the experts $\textsf {E}=\bigl \{E_1,E_2,E_3,E_4\bigr \}$ were combined into a *consensus standard* *S*, to be compared with all annotators $\textsf {A}$. Note that expert $E_0$ was not included in the consensus standard to avoid bias. To avoid an unfair advantage to the remaining experts, 4 different standards $S_{234},S_{134},S_{124},S_{123}$ were created, in each case excluding the expert being evaluated. Other annotators ($E_0,N_1,N_2,N_3,M$) were evaluated on these 4 consensus standards and the results averaged.

To create the consensus standard from the expert annotations $\textsf {B}_{ia}$ for an image *i* and $a\in \textsf {E}$, where $\textsf {E}$ is the set of experts involved, the following greedy algorithm was used, similar to the one in “Pairwise comparison” section

1. Find the largest box *b* from all $\textsf {B}_{i\tilde{a}}$, with $\tilde{a}\in \textsf {E}$. Remove *b* from $\textsf {B}_{i\tilde{a}}$.
2. For each $a'\in \textsf {E}$, $a'\not =\tilde{a}$, find boxes $b_{a'} \in \textsf {B}_{ia'}$ such that $b_{a'}\cong b$ (1), i.e., the boxes match. Let $\textsf {B}'$ be a set of such boxes $b_{a'}$, possibly empty.
3. Remove all boxes $\textsf {B}'$ from their original sets $\textsf {B}_{ia'}$.
4. If $\bigl |\textsf {B}'\bigr |+1>\bigl |\textsf {E}\bigr |/2$, take the coordinate-wise mean of the bounding boxes $\textsf {B}'\cup \{b\}$ and add the resulting mean bounding box to the consensus standard *S*.
5. Otherwise, add *b* to a minority set $S'$.
6. Repeat until all $\textsf {B}_{ia}$ are empty.

As a result, the consensus standard *S* contained lesions marked by the majority of experts (in our case two or three). Other lesions marked by a single expert were considered tentative and included in the minority set $S'$. Tentative lesions were counted as neither true positive nor false positive detections.

The resulting numbers of annotated lesions in the consensus standard are shown in Table 2. It can be seen that the agreement between experts was again weak, the number of unconfirmed lesions proposed by one of the experts was similar in scale to the number of lesions confirmed by the majority.

**Fig. 2:**
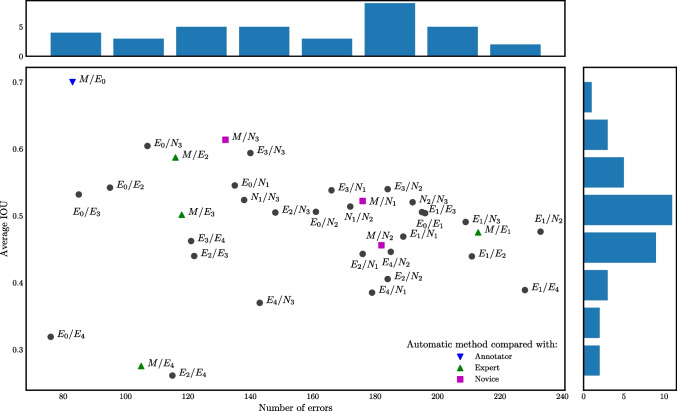
Pairwise agreement between annotators in terms of the number of errors $e_{aa'}$ (horizontally) and $\text {IoU}_{aa'}$ (vertically). Best agreement corresponds to the top left corner. The comparison with the automatic method is shown as color symbols, the comparison between human annotators is shown in black. Marginal histograms of $e_{aa'}$ and $\text {IoU}_{aa'}$ are shown at the top and right, respectively

Since expert $E_1$ seemed to annotate very differently from the other experts, having marked almost twice as many lesions (see Table 1), a reduced version of the standards was also created without expert $E_1$. In this case, consensus standards were created based on only two experts and both had to agree for a lesion to be included; otherwise, their annotations were considered as tentative.

**Table 2:** The number of annotated lesions agreed on by the majority of experts and by a single expert (minority) in the consensus standard. See Table 1 for comparison

	Number of annotations	
Consensus standard	Majority *S*	Minority $S'$
$S_{2,3,4}$	245	180
$S_{1,3,4}$	275	238
$S_{1,2,4}$	251	306
$S_{1,2,3}$	275	276

For each annotator, IoU and the number of errors *e* with respect to all applicable consensus standards were calculated and averaged over these standards. For results, see “Comparisonwith a consensus standard” section.

### Comparison with the original annotator

Finally, all annotators were compared with the original annotator $E_0$. Note that this may have favored *M*, which learned from $E_0$.

To evaluate statistical significance of the differences between annotators *a* and *b*, the Wilcoxon signed-rank test was applied to the sequence:

8 $$\begin{aligned} f^i_{ab}= e(\textsf {B}_{ia},\textsf {B}_{iE_0})-e(\textsf {B}_{ib},\textsf {B}_{iE_0}) \end{aligned}$$

and similarly for IoU. For results, see “Comparison with thethe original annotator” section.

**Table 3:** The pairwise differences in $\text {IoU}_{aa'}$ (above diagonal) and $e_{aa'}$ (below diagonal) for all pairs of annotators on the test dataset $D_1$, as well as the mean values of $\text {IoU}_{a}$ and $e_{a}$ in the last column and row, respectively, with best values in bold

	*M*	$E_0$	$E_1$	$E_2$	$E_3$	$E_4$	$N_1$	$N_2$	$N_3$	$\text {IoU}_a$
*M*	$\bullet$	0.7	0.48	0.6	0.51	0.27	0.52	0.45	0.62	0.52
$E_0$	89	$\bullet$	0.49	0.5	0.53	0.32	0.52	0.52	0.62	**0.53**
$E_1$	221	209	$\bullet$	0.44	0.51	0.39	0.47	0.48	0.49	0.47
$E_2$	120	148	211	$\bullet$	0.44	0.26	0.44	0.41	0.5	0.45
$E_3$	132	85	195	122	$\bullet$	0.46	0.54	0.54	0.59	0.52
$E_4$	111	76	228	115	121	$\bullet$	0.39	0.45	0.37	0.36
$N_1$	186	138	189	176	166	179	$\bullet$	0.51	0.52	0.49
$N_2$	184	192	233	184	184	185	172	$\bullet$	0.52	0.48
$N_3$	134	134	209	148	140	143	138	192	$\bullet$	**0.53**
$e_a$	147.1	**133.9**	211.9	153.0	143.1	144.8	168.0	190.8	154.8	$\bullet$

## Results

### Pairwise comparison

Two measures, $\text {IoU}_{aa'}$ and $e_{aa'}$ (“Pairwise comparison” section), are shown for all pairs of annotators in Fig. 2. It can be seen that the automatic method *M* was the closest to the original annotator $E_0$, and the comparisons of *M* with $E_2$, $E_3$, and $E_4$ are also well within the cloud of other pairwise comparison results, yielding very good results especially in terms of the number of errors *e*. The numeric values of $\text {IoU}_{aa'}$ and $e_{aa'}$ are presented in Table 3. Even the best matching annotators disagreed on 76 lesions, i.e., almost one false positive or false negative annotation per image. Perhaps surprisingly, two experts could disagree on more than 200 lesions in a dataset $D_1$ containing 100 images. Out of 5 experts, the automatic method outperformed 2 in terms of $e_a$ and 3 in terms of $\text {IoU}_a$.

**Fig. 3:**
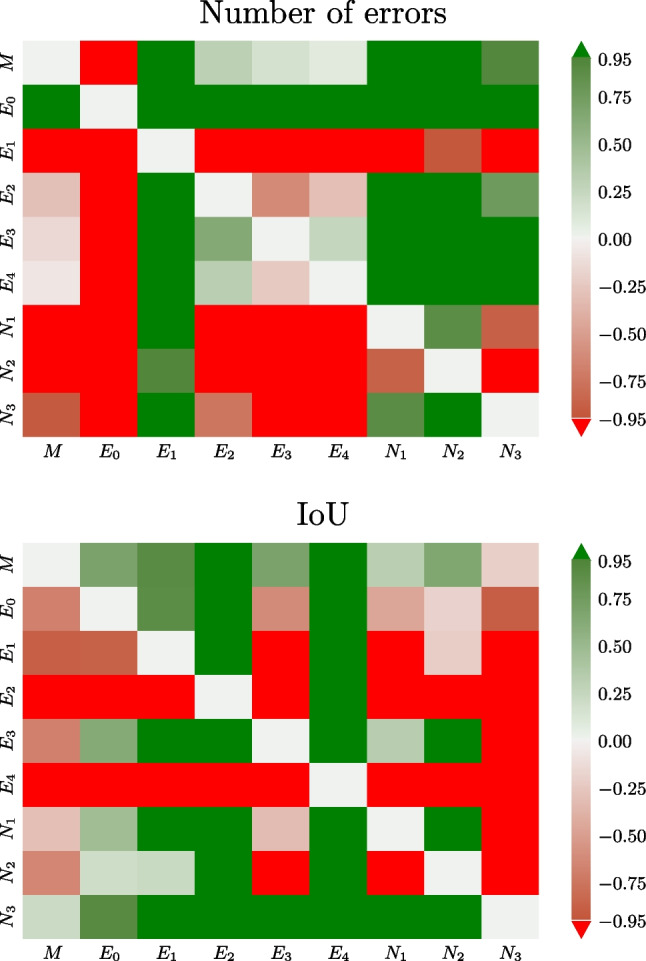
The quantity $q=\pm (1-p)$ from the Wilcoxon signed-rank test on the difference in the number of errors (top) and IoU (bottom) between an annotator and experts (see “Significance of pairwise differences” section). Green color (positive values) indicates that the row annotator is on the average closer to the experts than the column annotator and vice versa for red. Saturated green and red indicate statistically significant differences ($p<0.05$)

The statistical significance (at level $\alpha =0.05$ for all statistical tests) of pairwise differences between annotators according to the Wilcoxon test (“Significance of pairwisedifferences” section) is graphically displayed in Fig. 3. The automatic method *M* made significantly fewer errors than all the novices *N* and expert $E_1$ (Fig. 3, top). The number of errors made by *M* was also lower than that of $E_2$, $E_3$, and $E_4$ but not significantly so. In terms of the average IoU with respect to the experts (Fig. 3, bottom), the automatic method *M* was better than all other annotators except $N_3$. However, the difference was significant only for $E_2$ and $E_4$.

**Fig. 4:**
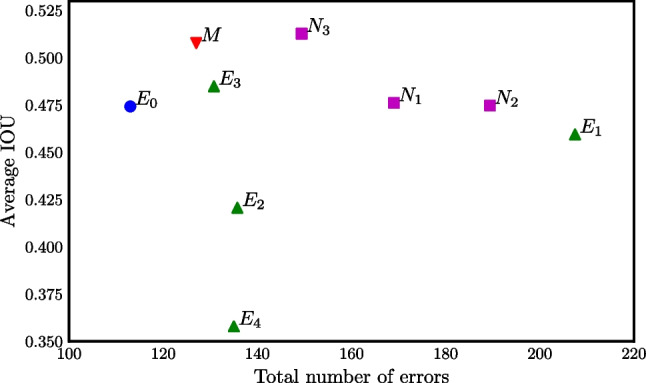
Mean $\text {IoU}_{a}$ and number of errors $e_{a}$ for all anotators averaged over experts different from *a*. An ideal result would be in the top left

The number of errors and IoU averaged over all other experts is shown in Fig. 4. It can be seen that the automatic method *M* is among the best two methods in terms of IoU with a minimal difference and second to only $E_0$ in terms of the number of errors *e*.

### Comparison with a consensus standard

Tables 4 and 5 present the outcome of comparisons with consensus standards, with and without expert $E_1$ (“Comparisonwith a consensus standard” section). In terms of the number of errors *e*, the automatic method *M* outperformed the novices $N_1$, $N_2$, $N_3$ and experts $E_1$, $E_2$, $E_4$ (Table 4). Excluding expert $E_1$ from the standards (Table 5), *M* outperformed all other annotators except $E_0$. In terms of IoU, no method reached very high values (compare with Fig. 2), the automatic method *M* being slightly below average.

**Table 4:** IoU and number of errors for all annotators *a* with respect to a consensus standard, created as a majority consensus of experts excluding $E_0$ and the expert being evaluated (shown by dashes)

	$S_{2,3,4}$		$S_{1,3,4}$		$S_{1,2,4}$		$S_{1,2,3}$		Average	
*a*	IoU	*e*	IoU	*e*	IoU	*e*	IoU	*E*	IoU	*e*
*M*	0.492	64	0.423	78	0.484	71	0.580	80	0.495	73
$E_0$	0.519	27	0.469	54	0.519	44	0.587	52	0.524	** 44**
$E_1$	0.530	136	—	—	—	—	—	—	0.530	136
$E_2$	—	—	0.382	84	—	—	—	—	0.382	84
$E_3$	—	—	—	—	0.595	59	—	—	**0.595**	59
$E_4$	—	—	—	—	—	—	0.390	90	0.390	90
$N_1$	0.557	101	0.526	82	0.548	74	0.559	81	0.547	85
$N_2$	0.573	122	0.557	117	0.556	106	0.534	114	0.555	115
$N_3$	0.580	78	0.519	78	0.548	71	0.603	80	0.563	77

Best average values are shown in bold

### Comparison with the original annotator

Using the original annotator $E_0$ as a reference (“Comparisonwith the original annotator” section), the automatic method was the best in terms of IoU and second best after $E_4$ in terms of the number of errors *e* (Table 6). The values of precision, recall and $F_1$ score for *M* were 0.78, 0.73 and 0.75, respectively.

**Fig. 5:**
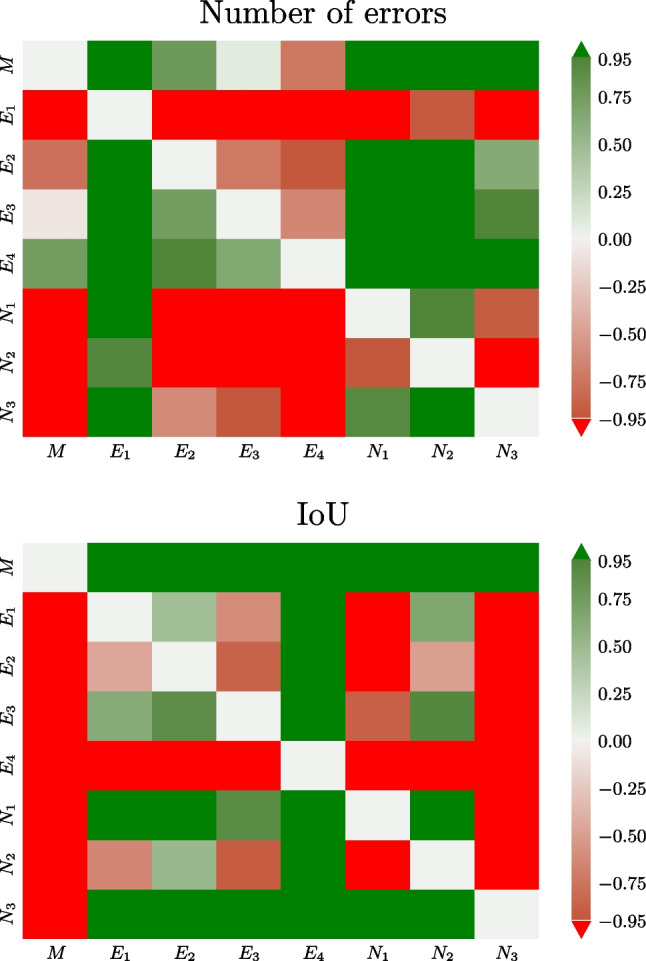
The quantity $q=\pm (1-p)$ from the Wilcoxon signed-rank test for the difference in the number of errors (top) and the IoU (bottom) between an annotator and expert $E_0$ on $D_1$ (see “Comparison with theoriginal annotator” section). Green color (positive value) indicates that the row annotator is on the average closer to $E_0$ than the column annotator and vice versa for red. Saturated green and red indicate significant changes ($q>0.95$ or $q<-0.95$, respectively)

The statistical significance of the differences between annotators with respect to $E_0$ is shown in Fig. 5. The automatic method *M* significantly outperformed all novices $N_1$, $N_2$, and $N_3$ in terms of the number of errors *e* (Fig. 5, top). It also outperformed experts $E_1$, $E_2$ and $E_3$ with the difference being significant only for $E_1$. In terms of IoU, the automatic method *M* was significantly closer to $E_0$ than all other annotators (Fig. 5, bottom). This was expected, since *M* learnt from $E_0$, but it nevertheless confirmed that the automatic method error is smaller than differences between experts.

**Table 5:** IoU and number of errors for all annotators *a* with respect to a consensus standard, created as a majority consensus of experts excluding $E_0,E_1$ and the expert being evaluated (shown by dashes)

	$S_{3,4}$		$S_{2,4}$		$S_{2,3}$		Average	
*a*	IoU	*e*	IoU	*e*	IoU	*e*	IoU	*e*
*M*	0.367	51	0.467	54	0.634	55	0.489	54
$E_0$	0.418	19	0.511	27	0.620	28	0.517	** 25**
$E_1$	0.487	150	0.539	161	0.546	141	0.524	151
$E_2$	0.341	59	—	—	—	—	0.341	59
$E_3$	—	—	0.621	62	—	—	**0.621**	62
$E_4$	—	—	—	—	0.376	57	0.376	57
$N_1$	0.484	111	0.55	119	0.565	109	0.533	113
$N_2$	0.533	122	0.581	126	0.544	123	0.553	124
$N_3$	0.492	80	0.566	88	0.645	82	0.568	83

Best average values are shown in bold

**Table 6:** Mean IoU and number of errors on the test dataset $D_1$ with respect to expert $E_0$

Measure	*M*	$E_1$	$E_2$	$E_3$	$E_4$	$N_1$	$N_2$	$N_3$
IoU	**0.523**	0.391	0.363	0.397	0.228	0.423	0.371	0.46
*e*	83	196	95	85	**76**	135	161	107

Best values marked in bold

## Discussion

In this study, the best performing automatic caries detection method from the companion paper Part I [17] was validated by a comprehensive comparison with human annotators, specifically four highly experienced dentists (experts), three novices with less than five years of experience, and the original annotator who created the training dataset. The comparison was performed on an independent dataset of 100 bitewing radiographs, and while it was expected that the annotations by individual annotators would differ, the difference was surprisingly high (see “Methods” section, Table 1, Fig. 1). This demonstrated the difficulty of defining the ground truth for an objective comparison. In other comparable (i.e., in vivo) studies, the reported inter-rater agreement on evaluating bitewing radiographs ranged between $\kappa =0.6$ in [19] to $\kappa =0.8$ in [20] and was even as low as $\kappa =0.246$ [16]. (Please note that this study formulates the task as a detection, not classification, so the absence of caries is not explicitly labeled and $\kappa$ cannot be calculated.)

Since the ground truth was not available, it was impossible to accurately measure the diagnostic performance of the automatic method. Consequently, multiple complementary methods were used for the evaluation.

The first approach consisted of pairwise comparisons between all annotators (“Pairwise comparison” section), including the automatic method. It was evaluated how many of their annotations matched, and non-matching annotations were considered errors. In this aspect, the automatic method was significantly outperformed only by the original annotator (see Fig. 3, top). The mean intersection over union (IoU, i.e., overlap) was generally low, the automatic method ranked among the best with IoU=0.52 (Table 1, Fig. 3, bottom).

However, pairwise comparisons have limitations, as they evaluate agreement rather than correctness. Therefore, the second approach was based on creating a consensus standard of the experts (“Comparison with a consensus standard” section), considering only lesions on which the majority of experts agreed. The automatic method was outperformed only by 2 of the 5 experts in terms of the number of errors (Table 4). The overlap (IoU) was again generally low for all annotators but the differences are probably not very meaningful, as the ability to detect caries in bitewing radiographs is clinically more important than slight variations in lesion size. The automatic method *M* was below average in terms of IoU. On the one hand, it was outperformed by the novices, on the other hand, some of the experts performed worse than *M*. This indicates the need to discuss a suitable IoU threshold for future studies on caries detection using deep learning. Note however, that our reported IoU are only calculated from matching annotations (as defined in “Pairwise comparison” section).

Finally, all annotators were compared with the original annotator $E_0$ (“Comparison with the original annotator” section). While this creates some advantage for the automatic method *M* that learnt from $E_0$, such biased approach is common in machine learning studies. The ground truth used for comparison with dentists is generally produced by the same expert(s) who have annotated the training dataset [13, 20, 21], only Bayrakdar et al. [22] invited two additional experts to annotate the test dataset. In this study, the automatic method made fewer errors than all dentists except $E_3$, and it was the best in terms of the average IoU by a significant margin (Table 6), showing that it learnt the annotation style of $E_0$ well. Even so, there were 83 differences (errors) between *M* and $E_0$ on the dataset $D_1$. This number may seem high but given that the average of 13 proximal surfaces per radiograph in the test dataset, the 83 errors correspond to a classification error of $83/(100\cdot 13)=6.4\%$. Moreover, only one of the experts achieved a smaller value. The detection performance corresponds to an $F_1$ score of 0.75 (“Comparison withthe original annotator”) which is lower than $F_1=0.80$ on the training dataset $D_0$ [17]. This may have been caused by a slightly higher prevalence of caries in the $D_1$ dataset or an inconsistence of annotations of the expert $E_0$, as $D_1$ was annotated approximately 6 months after $D_0$.

It is also noteworthy that the datasets $D_0$ and $D_1$ contained radiographs acquired using several different intraoral X-ray machines and sensors. This increases both the variability of the dataset and the difficulty of correct detection for the automatic method, thus possibly decreasing the detection accuracy. On the other hand, a model trained on such data should generalize better and perform well also for other unseen variants of bitewings radiographs. Overall, the results of the automatic model were fully comparable with experienced dentists. It seems that further improvement will require a new approach to determine a reliable ground truth.

## Conclusions

Repeatable and accurate caries detection in bitewing radiographs is challenging even for experienced dentists, which was confirmed by the marked differences between expert annotators. The tested automatic method consistently outperformed novices, and its performance was similar or superior to highly experienced experts. The presented method could therefore provide a useful second opinion for dentists, especially those with limited clinical experience, and help in improving both the accuracy and repeatability of caries detection.

## Notation and abbreviations

$\bigl |\cdot \bigr |$: Number of elements in a set
*a*,$\textsf {A}$: Annotator, set of all annotators
*b*, $b\cong b'$: Bounding box, matching boxes
$\textsf {B}$: Set of bounding boxes
$$D_0$$: Training dataset
$$D_1$$: Test dataset
*e*: Number of annotation errors
*E*,$\textsf {E}$: Expert annotator, set of expert annotators
*i*: Image
*M*: Automatic method
*N*: Novice annotators
*S*: Consensus standard
$\kappa$: Cohen’s kappa coefficient of inter-rater reliability
$\Omega$: Matching between two sets of bounding boxes
CNN: Convolutional neural network
CVAT: Computer vision annotation tool
IoU: Intersection over union
R-CNN: Region-based object detection architecture
ResNet: Residual neural network (architecture)
RetinaNet: CNN object detection architecture
*Swin*: Shifted windows (transformer architecture)
*YOLO*: You only look once (object detection architecture)

## Footnotes

1 https://github.com/opencv/cvat
